# Intracellular Kinases Mediate Increased Translation and Secretion of Netrin-1 from Renal Tubular Epithelial Cells

**DOI:** 10.1371/journal.pone.0026776

**Published:** 2011-10-26

**Authors:** Calpurnia Jayakumar, Riyaz Mohamed, Punithavathi Vilapakkam Ranganathan, Ganesan Ramesh

**Affiliations:** Vascular Biology Center, Georgia Health Sciences University, Augusta, Georgia, United States of America; National Cancer Institute, United States of America

## Abstract

**Background:**

Netrin-1 is a laminin-related secreted protein, is highly induced after tissue injury, and may serve as a marker of injury. However, the regulation of netrin-1 production is not unknown. Current study was carried out in mouse and mouse kidney cell line (TKPTS) to determine the signaling pathways that regulate netrin-1 production in response to injury.

**Methods and Principal Findings:**

Ischemia reperfusion injury of the kidney was induced in mice by clamping renal pedicle for 30 minutes. Cellular stress was induced in mouse proximal tubular epithelial cell line by treating with pervanadate, cisplatin, lipopolysaccharide, glucose or hypoxia followed by reoxygenation. Netrin-1 expression was quantified by real time RT-PCR and protein production was quantified using an ELISA kit. Cellular stress induced a large increase in netrin-1 production without increase in transcription of netrin-1 gene. Mitogen activated protein kinase, ERK mediates the drug induced netrin-1 mRNA translation increase without altering mRNA stability.

**Conclusion:**

Our results suggest that netrin-1 expression is suppressed at the translational level and MAPK activation leads to rapid translation of netrin-1 mRNA in the kidney tubular epithelial cells.

## Introduction

Netrin-1 is a laminin-related secreted protein that is widely expressed in many tissues, including kidney [1], [2]. Netrin-1 was shown to regulate neuronal migration [3], inflammation during ischemia reperfusion injury of the kidney [1], [4], [5], lung [6], whole animal hypoxia [7], cisplatin induced kidney injury [8], angiogenesis during development [9], and in adult heart [10]. In addition, netrin-1 is also shown to increase kidney epithelial proliferation and migration [11] and cancer development and progression [12], [13]. Netrin-1 is known to bind to three distinct families of receptors, the DCC family (DCC and neogenin), the UNC5 family (UNC5A-D), and DSCAM, to mediate it biological effects in different tissues. The highest levels of netrin-1 mRNA were seen in the kidney, among the many organs studied so far; however netrin-1 protein expression is minimal in the kidney [1], [2]. Localization studies had determined that netrin-1 expression is restricted to vascular endothelial cells, and little or no expression was seen in the tubular epithelial cells. However, within hours after injury of the tubules, netrin-1 protein is induced and excreted into urine [14], [15]. Therefore, netrin-1 was identified as an early diagnostic biomarker of acute kidney injury (AKI) in mice as well as in humans [14], [16]. However, the mechanism through which renal insult induced netrin-1 protein expression was unknown. Clinically AKI is characterized by a rapid reduction in kidney function resulting in a failure to maintain fluid, electrolyte and acid-base homoeostasis [17]. There are many causes of acute kidney injury in human which may include, ischemia of the kidney, drug administration such as cisplatin and gentamycin, and infection.

We investigated the regulation of netrin-1 at the transcriptional and translational levels in mouse kidney tubular epithelial cells in vitro. Pervanadate was used to induce as oxidative stress and MAPK activation. Pervanadate is a powerful phosphatase inhibitor that leads to the accumulation of phosphorylated proteins and the activation of kinases that are normally retained in inactive forms by dephosphorylation [18], [19]. Our results showed that netrin-1 is rapidly translated in tubular epithelial cells, which is regulated by stress-activated MAPK pathways.

## Materials and Methods

### Renal ischemia reperfusion

C57BL/6J mice (8–9 weeks of age, The Jackson Lab, Bar Harbor, ME) were anesthetized with sodium pentobarbital (50 mg/kg body weight, intra-Peritoneally) and were placed on a heating pad to maintain body temperature at 37°C. Both renal pedicles were identified through dorsal incisions and clamped for 30 minutes. Reperfusion was confirmed visually upon release of the clamps. As a control, sham-operated animals were subjected to the same surgical procedure except the renal pedicles were not clamped. Surgical wounds were closed and mice were given 1 ml of warm saline, intraperitoneally. The mice were kept in a warm incubator until they regained consciousness. Urine and kidney tissue were collected 6 hrs after reperfusion and processed for ELISA and RNA isolation. The institutional animal care and use committee of the Georgia Health Sciences University approved all of the protocols and procedures using animals and the approval number is BR10-10-369.

### Cell culture

Cultured murine proximal tubule cells (TKPT cells, kindly provided by Dr. E. Bello-Reuss, University of Texas Medical Branch, Galveston, TX) were cultured in advanced DMEM/F12 medium supplemented with glutamine, 7.5% FBS and antibiotics. Cells were grown to confluence and maintained at 37°C in 5% CO_2_ [20]. All experiments were carried out in serum free DMEM/F12 medium. Cells were in the serum free advanced DMEM/F12 medium for a minimum period of 1 hour and maximum of 24 hours during experimentation. No significant increase in apoptosis was seen in serum free medium within 24 hours of culture. Cells were treated with different concentrations of pervanadate for the indicated times and then were harvested for Western blot and RNA isolation. When indicated, cells were incubated with inhibitors of p38 MAP kinase (10 µM SB203580), ERK (10 µM U0126), or JNK (20 µM SP600125, from Celgene, Signal Research Division, San Diego, CA) for 30 min before pervanadate addition. The concentration of MAPK inhibitors were chosen based on our previous studies in TKPTS cell line [20]. To examine the effect of pervanadate on netrin-1 mRNA stability, 10 µg/ml actinomycin D was added 10 minutes after pervanadate addition. To examine the influence of translational arrest on mRNA stability, cycloheximide was added at a concentration of 7.5 µg/ml and cells were harvested at different time points. Pervanadate was prepared by mixing equimolar quantities of hydrogen peroxide and sodium orthovanadate and used within 15 minutes of preparation.

### RNA isolation

Total RNA was isolated using TRIZOL reagent (Invitrogen, Carlsbad, CA). Briefly, kidney was homogenized in 2 ml of TRIZOL reagent (20 ml/g of tissue weight). Tissue homogenate was spun at 10,000 g for 10 minutes to remove insoluble materials. Homogenate was vortexed thoroughly after adding 400 µl of chloroform and spun again at 10,000 g for 20 minutes. Supernatant was transferred and RNA was precipitated by adding equal volume of isopropanol. Total RNA was isolated from TKPTS cells by lysing cells in 400 µl of Trizol and then vortexted after adding 100 µl of chloroform. Homogenate was spun at 10000 g for 20 minutes at 4 C. Supernatant was transferred to another tube and RNA was precipitated by adding equal volume of isopropanol. RNA pellet was dissolved in sterile nuclease free water and then quantified using spectrophotometer. 260/280 ratio for RNA measurement was between 1.9 to 2.0.

### Western blot analysis

Cells were scraped from the dish and centrifuged at 10,000 g for 5 min. The cell pellet was solubilized in RIPA buffer containing protease and phosphatase inhibitor cocktail. The protein concentration was measured using the BCA protein assay reagent (Pierce). 100 µg aliquots of total protein were resolved in 10% polyacrylamide gels and then transferred to a PVDF membrane. The membrane was probed with phospho-specific antibodies to p38 MAPK, ERK and JNK (Cell Signaling Technologies, Beverly, CA). Proteins were detected using enhanced chemiluminescence detection reagents.

### Quantitation of mRNA by real time PCR

Real time PCR was performed in an Applied Biosystem Inc 7700-sequence detection system. 1 microgram of total RNA was reverse transcribed in a reaction volume of 20 µl using Superscript II reverse transcriptase and random primers. The product was diluted to a volume of 50 µl and 5-µl aliquots were used as templates for amplification using SYBR Green PCR amplification reagent and gene specific primers. The primer sets used were: actin (forward: CATGGATGACGATATCGCT; reverse: CATGAGGTAGTCTGTCAGGT), netrin-1 (forward: AAGCCTATCACCCACCGGAAG; reverse: GCGCCACAGGAATCTTGATGC). The amount of RNA was normalized to the actin signal amplified in a separate reaction.

### Netrin-1 quantification by ELISA

The level of netrin-1 in culture supernatant was quantified using an ELISA assay (Mouse netrin-1 ELISA kit, USCNk Life Sciences Inc., Wuhan, China) according to the manufacturer's instructions.

### *In vitro* hypoxia and reoxygenation

TKPTS cells were subjected to hypoxia using hypoxic bags (BD Biosciences) for 2 hrs and then followed by reoxygenation for 2, 4 and 24 hrs. Experiment was carried out in serum free advanced DMEM/F12 medium. Cells and supernatant were harvested for mRNA isolation, RT-PCR analysis, and quantification of netrin-1 by ELISA.

### Treatment of TKPTS cells with drugs

TKPTS cells at 90% confluence medium was replaced with serum free low glucose DMEM medium. Glucose (100, 400, 600 and 800 mg/dl), mannitol (600 and 800 mg/dl), cisplatin (50 µM), BSA (20 mg/ml) and lipopolysaccharide (1 µg/ml) were added. To determine the influence of ERK MAPK on drug-induced netrin-1 production in TKPTS cells, MEK2 specific inhibitor (10 µM) was added along with drugs. Cells and medium were collected at different times after addition of drugs and processed for RNA and ELISA.

### Statistical methods

All assays were performed in duplicate. The data are reported as means ± SEM. Statistical significance was assessed by unpaired, two-tailed Student's *t-*test for single comparison or ANOVA for multiple comparisons.

## Results

### Ischemia reperfusion induced increased secretion of netrin-1 from kidney, which is associated with down-regulation of netrin-1 mRNA

**Figure 1:**
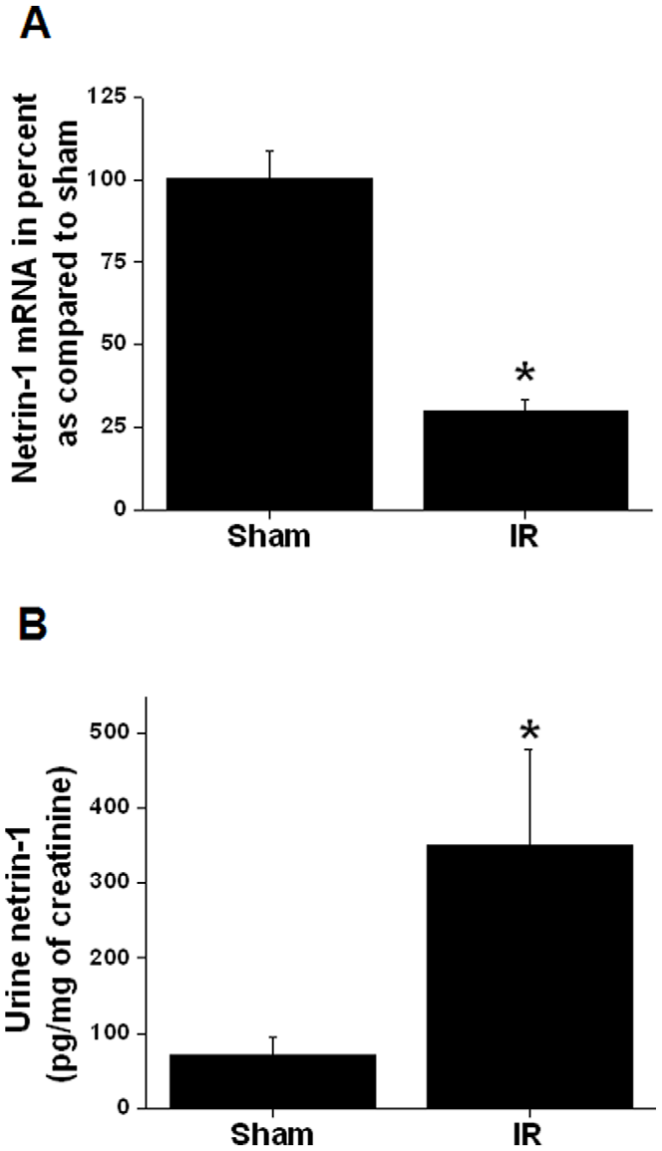
Netrin-1 mRNA and protein levels in kidney in response to ischemia reperfusion of the kidney. A. Netrin-1 mRNA levels in the kidney at 6 hr after reperfusion in ischemic and sham-operated animals. n = 4, *p<0.001 vs. sham-operated. B. Quantification of netrin-1 in urine at 6 hrs after reperfusion. Netrin-1 levels were normalized to per mg of urine creatinine. n = 4. *, *p*<0.001 vs. sham-operated animals.

Our earlier studies identified netrin-1 as an early diagnostic biomarker of acute kidney injury. Netrin-1 protein was highly induced after many forms of renal injury and excreted in urine. However, the regulation of netrin-1 expression is unknown. To determine whether netrin-1 induction is due to increase in transcription, mice were subjected to 30 minutes of ischemia followed by 3 and 6 hrs of reperfusion. Excretion of netrin-1 protein in urine and mRNA expression in kidney were quantified. As shown in Figure 1, ischemia reperfusion induced a large increase in netrin-1 excretion in urine at 3 hrs (not shown) and 6 hrs after reperfusion. However, netrin-1 mRNA levels were significantly reduced at 6 hrs after reperfusion, suggesting that increased secretion of netrin-1 was associated with rapid degradation of netrin-1 mRNA in tubular epithelial cells.

### Pervanadate increases netrin-1 production in renal proximal tubule cells

**Figure 2:**
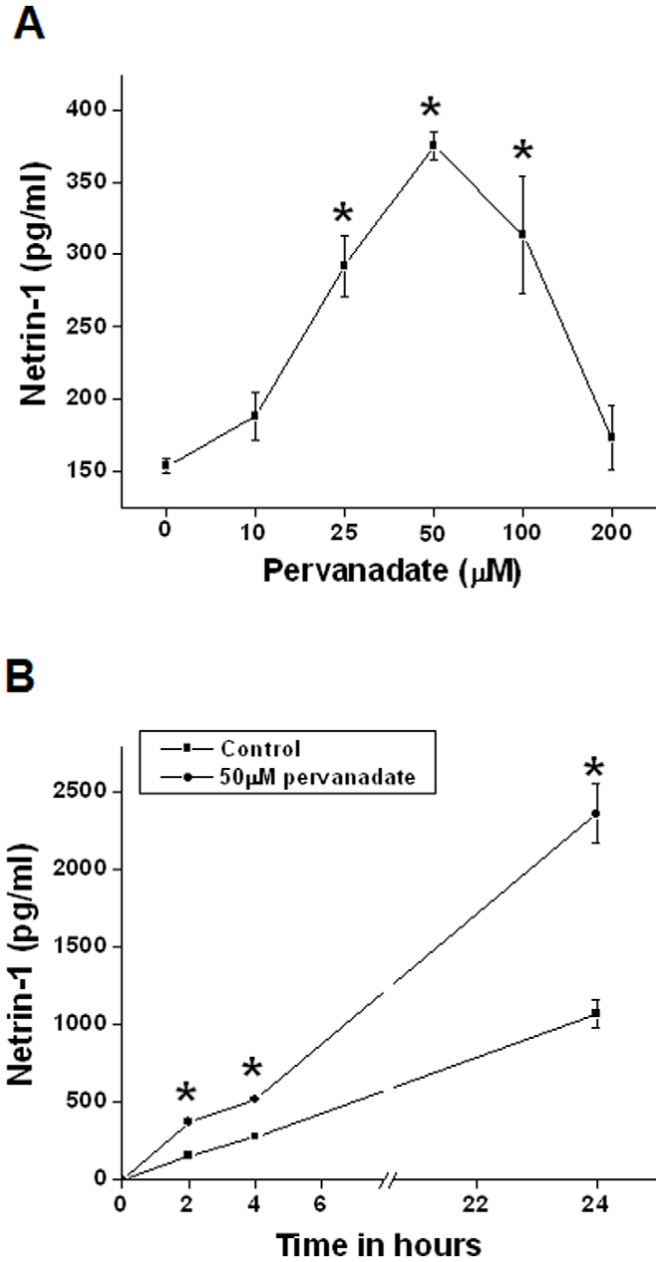
Pervanadate induces netrin-1 production in a dose- and time-dependent manner in TKPTS cells. A. Netrin-1 production at different doses of pervanadate. Cell supernatant was harvested at 2 hr after pervanadate addition. *p<0.001 vs. control. B. Netrin-1 production at different times after 50 µM pervanadate or vehicle addition. *, *p*<0.05 vs. vehicle-treated (control) group. n = 6.

As seen in Figure 1, netrin-1 excretion is increased in urine within hours after reperfusion but the expression of kidney netrin-1 mRNA is down-regulated. However the mechanism for increased netrin-1 production was unknown. Since oxidative stress and MAPK activation are prominent events after ischemia reperfusion and other forms of acute kidney injury, we used pervanadate to investigate the influence of cellular stress on the netrin-1 production in mouse proximal tubular epithelial cells (TKPTS). As shown in Figure 2, addition of pervanadate increased netrin-1 protein expression in a concentration-dependent and time-dependent manner.

### Pervanadate activates MAPKs in renal proximal tubule cells

**Figure 3:**
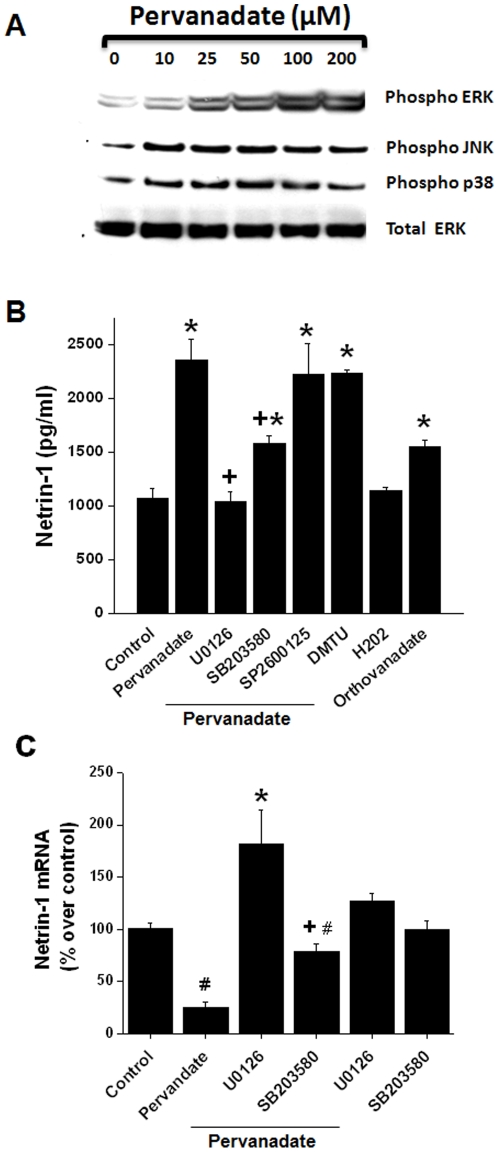
MAPK pathway mediates pervanadate-induced increase in netrin-1 production in TKPTS cells. A. Pervanadate increases the activation of MAPKs (ERK, p38 and JNK) as seen by increased phosphorylation with increasing dose of pervanadate. B. Pervanadate-induced increase in netrin-1 protein is suppressed in the presence of U0126 and to some extent SB203580 but not with SP2600125 and antioxidant. *, *p*<0.001 vs. control. +, *p*<0.001 vs. pervanadate alone treated group. n = 6. C. Effect of pervanadate and MAPK pathway inhibitors on netrin-1 transcript levels. Pervandate significantly decreased netrin-1 mRNA levels. In the presence of U0126 netrin-1 mRNA levels significantly increased. *, *p*<0.001 vs. pervanadate. #, *p*<0.05 vs. control. +, *p*<0.05 vs. pervanadate+U0126. n = 4.

Pervanadate is known to activate MAP kinases in HeLa and smooth muscle cells [18], [19]. Since MAP kinases are involved in the regulation of translation [20], we examined the effect of pervanadate on the activity of the three MAP kinase pathways in TKPTS cells by Western blot analysis. As shown in Figure 3A, pervanadate increased the phosphorylation of all three MAPKs in a concentration-dependent manner.

### Inhibition of MAP kinases reduces pervanadate-induced netrin-1 production

Pervanadate increases both netrin-1 expression (Figure 2) and MAPK activity (Figure 3A) in TKPTS cells. To determine if the activation of MAPK pathways mediates the increase in netrin-1 expression, TKPTS cells were treated with 50 µM pervanadate in the presence or absence of specific inhibitors of p38 MAP kinase (10 µM SB203580), ERK (10 µM U0126), or JNK (20 µM SP600125). As shown in Figure 3B, pervanadate induced a 2-fold increase in the secretion of netrin-1 protein. Inhibition of p38 and ERK reduced netrin-1 protein to near control levels. The JNK inhibitor SP600125 did not reduce netrin-1 protein levels. Interestingly, addition of the antioxidant dimethyl thiourea did not inhibit pervanadate-induced netrin-1 production, suggesting that netrin-1 production is via oxidative stress-independent mechanism. Also, hydrogen peroxide alone did not significantly alter the netrin-1 production whereas orthovanadate alone induced a marginal increase in netrin-1 production (Figure 3B).

To determine if the inhibition of MAP kinase inhibitors is due to an alteration in netrin-1 mRNA levels, netrin-1 transcripts were quantified in the presence of MAP kinase inhibitors. As shown in Figure 3C, pervanadate significantly reduced netrin-1 mRNA as compared to control. Addition of the MEK2 inhibitor U0126 suppressed pervanadate-induced netrin-1 degradation and significantly increased the amount of netrin-1 mRNA as compared to control. Addition of p38 MAP kinase inhibitor partially prevented pervanadate-induced netrin-1 mRNA degradation. Inhibitors alone did not affect netrin-1 mRNA nor protein levels. These results suggest that the ERK-mediated translation increase is also associated with enhanced degradation of netrin-1 mRNA in tubular epithelial cells.

### Pervanadate does not increase netrin-1 mRNA stability

**Figure 4:**
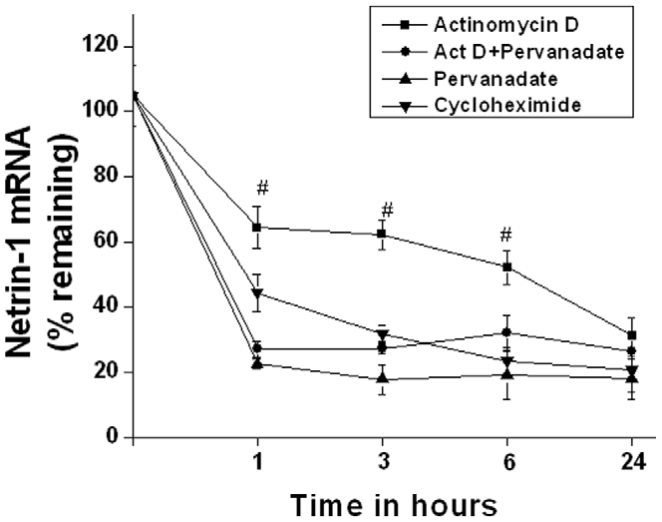
Pervanadate did not increase netrin-1 mRNA stability. mRNA degradation was quantified by real time RT-PCR at various times after addition of actinomycin D, actinomycin+pervanadate, pervanadate or cycloheximide. Addition of actinomycin D induced a rapid degradation of netrin-1 mRNA, which was further enhanced in the presence of pervanadate. Similarly, inhibition of translation also increased rapid netrin-1 mRNA degradation. #, *p*<0.05 vs. all other groups.

The expression levels of many secreted proteins are determined, in part, by their regulated degradation. We examined if the pervanadate-induced increase in netrin-1 protein was related to a stabilization of netrin-1 mRNA. The results in Figure 3 suggested that addition of pervanadate may enhance degradation of netrin-1 mRNA. To examine this issue more directly, we determined the effect of pervanadate on the degradation of netrin-1 mRNA in TKPTS cells. Cells were treated with pervanadate and/or 10 µg/ml actinomycin D was added to arrest further transcription. The levels of netrin-1 were determined at discrete time points over the next 24 hours (Figure 4). In cells treated with actinomycin D alone, netrin-1 mRNA decayed modestly, to ∼60% of original levels, by 60 minutes. In contrast, netrin-1 mRNA levels decayed rapidly, to ∼20% of the orginal levels by 60 minutes in cells treated with actinomycin D and pervanadate. These results indicate that pervanadate did not increase mRNA stability, thereby increasing netrin-1 production. Rather, pervanadate may affect translation of netrin-1 mRNA.

### Suppression of new protein synthesis enhances netrin-1 mRNA degradation

Stabilization and/or transcription of netrin-1 mRNA may involve the binding of certain proteins to the mRNA or gene. To determine if the slow degradation of netrin-1 with actinomycin D involves the synthesis of new protein, RNA stability was studied after translational arrest. TKPTS cells were treated with cycloheximide (Figure 4). In cells that received only actinomycin D, netrin-1 mRNA levels decay at a modest rate whereas in cycloheximide-treated cells, netrin-1 mRNA decay was enhanced, suggesting that new protein synthesis is required for the slow degradation of mRNA and/or transcription.

### Drug-induced increase in netrin-1 secretion is also mediated by ERK MAPK

**Figure 5:**
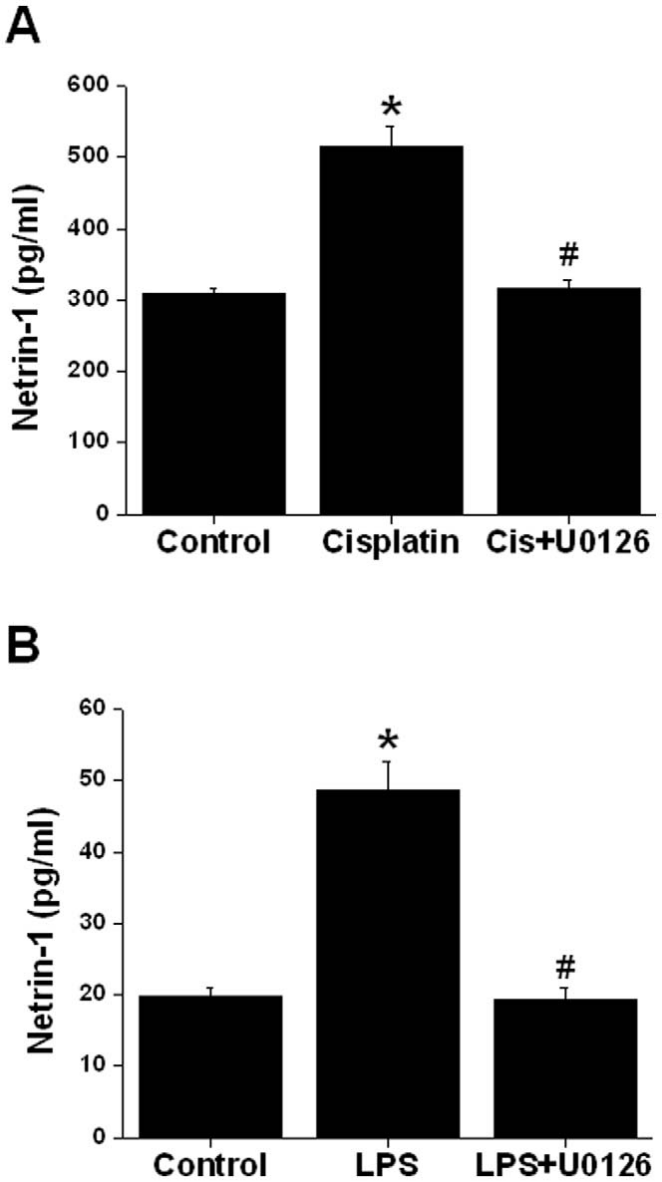
Drug-induced increase in netrin-1 production is also mediated by ERK MAPK pathway. A. 50 uM cisplatin was added to confluent culture with/without U0126. Six hours after addition supernatant was harvested. Netrin-1 was quantified by ELISA. Cisplatin significantly increased netrin-1 production, which was suppressed in the presence of U0126. *, *p*<0.001 vs. control. #, *p*<0.001 vs. cisplatin. n = 4. B. Lipopolysaccharide (µg/ml) was added to confluent culture with/without U0126. Two hours after addition, supernatant was harvested. Netrin-1 was quantified by ELISA. Lipopolysaccharide significantly increased netrin-1 production, which was suppressed in the presence of U0126. *, *p*<0.001 vs. control. #, *p*<0.001 vs. LPS. n = 4.

Our *in vivo* study shows that drugs, such as endotoxin and cisplatin that are known to induce acute kidney injury increased netrin-1 production from kidney tubules and are excreted in the urine [16]. However, the regulation of enhanced netrin-1 production was unknown. To determine the mechanism of drug induced netrin-1 production, TKPTS cells were treated with endotoxin and cisplatin as described in Materials and Methods. As shown in Figure 5a, cisplatin induced increased netrin-1 secretion significantly by 6 hr, whereas endotoxin-induced increase occurs in 2 hr. Addition of MEK2 inhibitor U0126 inhibited both the cisplatin and endotoxin-induced increase in netrin-1 production.

### Hyperglycemia suppresses netrin-1 secretion but high protein induces netrin-1 secretion through ERK MAPK

**Figure 6:**
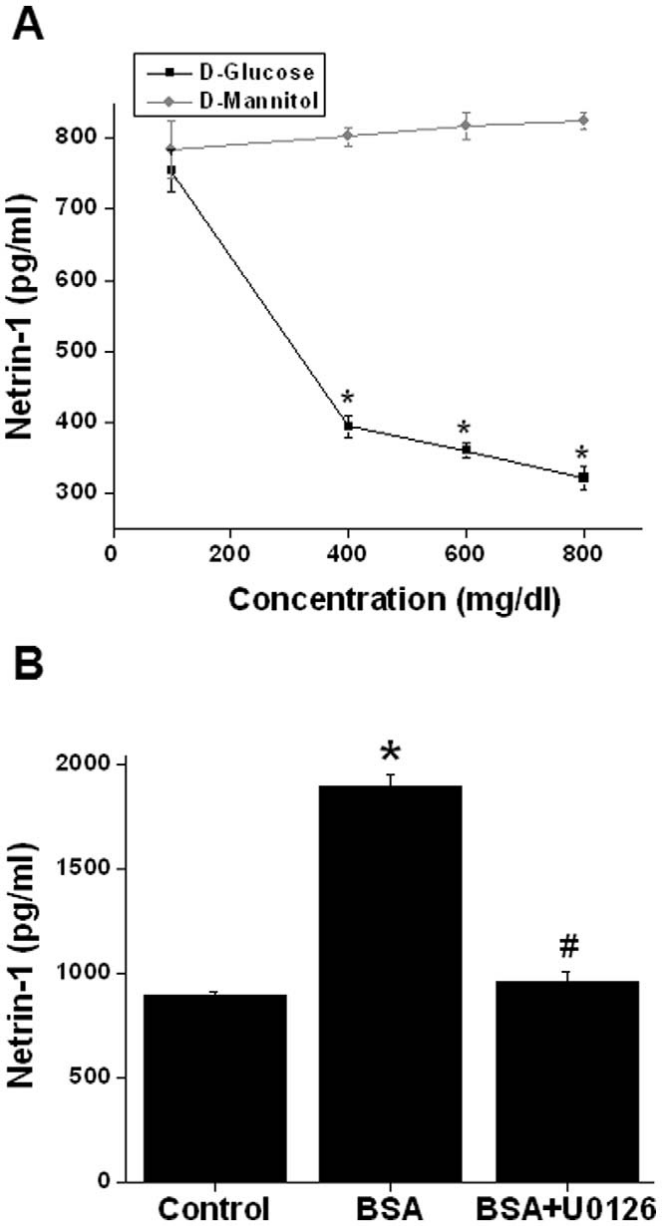
Regulation of netrin-1 production by glucose and protein (albumin) in TKPTS cells. A. Increasing concentration of D-glucose or mannitol was added to confluent culture. Twenty-four hours after addition the supernatant was harvested. Netrin-1 was quantified by ELISA. D-glucose down-regulated netrin-1 production in a dose-dependent manner whereas D mannitol did not alter netrin-1 production. *, *p*<0.001 vs. mannitol group. n = 4. B. Bovine serum albumin (BSA) (20 mg/ml) was added to confluent culture with/without U0126. Twenty-four hours after addition supernatant was harvested. Netrin-1 was quantified by ELISA. BSA significantly increased netrin-1 production, which was suppressed in the presence of U0126. *, *p*<0.001 vs. control. #, *p*<0.001 vs. BSA. n = 6.

Like acute kidney injury, chronic kidney diseases such as diabetic nephropathy also show enhanced production of netrin-1 and increased levels in urine (unpublished observation). However, whether hyperglycemia itself can induce enhanced netrin-1 production from renal tubular epithelial cells is unknown. To determine whether hyperglycemia induces netrin-1, an increased concentration of glucose was added to tubular epithelial cell cultures. As shown in Figure 6A, increased glucose concentration has an opposite effect on netrin-1 production, suggesting that hyperglycemia itself is not a signal for the enhanced netrin-1 production seen *in vivo*. Mannitol alone did not have any influence on netrin-1 production.

Diabetic nephropathy is associated with increased proteinuria which may induce netrin-1 production. To determine whether increased amount of protein can induce netrin-1, TKPTS cells were treated with different concentrations of albumin (low endotoxin and fatty acid free) for 24 hrs. As shown in Figure 6B, increased albumin concentration enhanced netrin-1 production, which was completely suppressed by MEK2 inhibitors, suggesting that ERK MAPK mediates albumin-induced netrin-1 production.

### Hypoxia and reoxygenation-induced increase in netrin-1 production is also mediated by ERK MAPK

**Figure 7:**
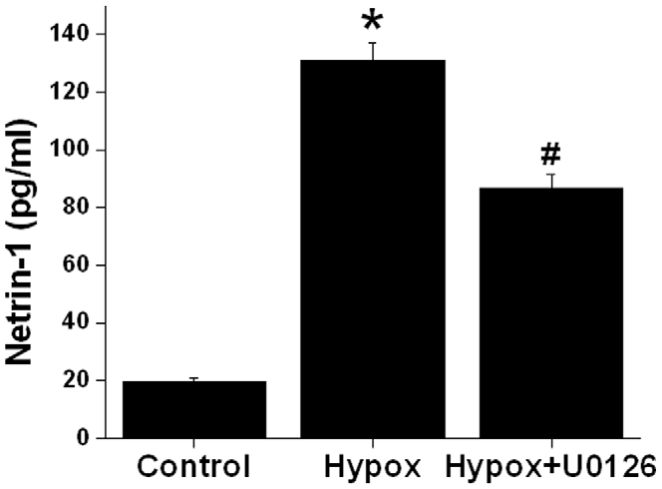
Hypoxia reoxygenation-increased netrin-1 secretion in TKPTS cells is mediated by ERK MAPK pathway. Confluent TKPTS cells were subjected to hypoxia followed by 2 hr of reoxygenation and supernatant was then harvested. Netrin-1 was quantified by ELISA. Hypoxia and reoxygenation significantly increased netrin-1 production which was suppressed in the presence of U0126. *, *p*<0.001 vs. control. #, *p*<0.001 vs. cisplatin. n = 6.

As shown Figure 1, ischemia reperfusion induced increased excretion of netrin-1 in urine. However, the mechanism was unknown. To determine the role of intracellular kinase in enhanced production of netrin-1, TKPTS cells were subjected to hypoxia and reoxygenation as described in Materials and Methods. As shown in Figure 7, hypoxia reoxygenation increased netrin-1 protein secretion, which was significantly inhibited by MEK2 inhibitor U0126.

## Discussion

Netrin-1 is an early diagnostic biomarker of acute kidney injury and cancers [14], [15], [21]. In the kidney, netrin-1 protein is mostly expressed in vascular endothelial cells. In response to ischemia reperfusion, endothelial expression is down-regulated and tubular epithelial expression is up-regulated. However, the mechanism of regulation of netrin-1 production during injury of the kidney or other tissue was unknown. In this report, we examined the mechanism of netrin-1 production in mouse proximal tubular epithelial cells (TKPTS) *in vitro* and ischemia reperfusion injury of the kidney *in vivo*. Our results show that netrin-1 protein is highly induced but the induction is not due to increased transcription of netrin-1 gene. Rather, netrin-1 expression is regulated at the translational level by MAPKs, independent of mRNA stability. An ERK mediated increase in netrin-1 production was also seen *in vitro* with drugs that are known to cause acute kidney injury.

Our *in vivo* studies show a disconnect between increased protein and mRNA levels, suggesting that netrin-1 expression may be regulated at the posttranslational level. Translation of mRNA is regulated by several pathways. One of the important pathways is mitogen activated protein kinases (MAPK). Pervanadate is known to increase MAPK activation and suppresses phosphatase activity. Consistent with previous studies [18], [19], pervanadate addition to renal tubular epithelial cells increased the activation of all three MAP kinases (ERK, P38 and JNK). Moreover, pervanadate increased the secretion of netrin-1. We also showed that inhibition of MAPK pathways suppressed the pervanadate-induced increase in netrin-1 production. However, only ERK the and to some extent p38 pathways play a role in netrin-1 production whereas JNK inhibition did not alter the pervanadate-induced increase in netrin-1 production. The ERK pathway inhibitor reduced netrin-1 production more than p38 inhibitors. These results suggest the pervanadate-mediated increase in netrin-1 production in renal epithelial cells is mediated by the ERK and p38 pathways. To our knowledge, this is the first study to document the role of MAPK in netrin-1 production.

Pervanadate addition did not increase netrin-1 mRNA levels in TKPTS cells suggesting that increased production of netrin-1 in renal epithelial cells is not due to increased transcription. Therefore, MAPK may affect netrin-1 production at posttranscriptional levels. The stability of mRNA is an important element in the regulation of certain genes, including many involved in inflammatory responses [20]. Netrin-1 mRNA may be inherently unstable, which is supported by our data that either suppression of transcription or translation induced a rapid degradation of netrin-1 mRNA. Moreover, we determined that pervanadate enhanced the degradation of netrin-1 mRNA in TKPTS cells. These results suggest that the pervandate-induced increase in netrin-1 production is not due to increased netrin-1 mRNA stability.

The dual role of pervanadate or pervanadate-induced signaling in netrin-1 mRNA degradation while simultaneously increasing netrin-1 protein production is puzzling. Moreover, addition of the MAPK inhibitor U0126 suppressed netrin-1 protein production but increased netrin-1 mRNA content. It is possible that the ERK pathway may promote both translation and degradation of netrin-1 mRNA. Therefore, inhibition of ERK pathways leads to accumulation of netrin-1 mRNA. However, it is also possible that rapid translation of netrin-1 mRNA may be associated with rapid degradation of netrin-1 mRNA in tubular epithelial cells independent of the ERK pathway. Our *in vivo* studies also support this view that increased excretion of netrin-1 protein after ischemia reperfusion injury was associated with a reduction in netrin-1 mRNA in the kidney. The mechanism for this opposing effect on protein and mRNA is not clear. Whether, the 3′ and 5′ untranslated regions of mRNA play a role in translation and stability is unclear. Scanning of 5′ and 3′ untranslated regions (UTRs) of netrin-1 mRNA show two unique sequences that are known to regulate translation and mRNA stability: an internal ribosome entry site (IRES) in the 5′ UTR and a K-box motif in the 3′ UTR. The role of these sequences in the regulation of netrin-1 translation and degradation is unknown and needs further study. To our knowledge, this is the first example of increased translation associated with increased degradion of mRNA in response to stress and hypoxia.

Our studies also show that the pervanadate-induced increase in translation of netrin-1 mRNA is not an artificial stress signal. The agents or stimuli that are known to induce acute kidney injury and the associated increase in netrin-1 *in vivo* also induced netrin-1 *in vitro* in TKPTS cells, which was again mediated through ERK MAPK pathways. In addition, drugs or hypoxia induced an increase in netrin-1 production without increasing netrin-1 transcription levels, significantly suggesting that regulation is at the translational level (data not shown).

In summary, we have determined that pervanadate, cisplatin, lipopolysaccharide (LPS), high protein levels and hypoxia induce netrin-1 secretion from proximal tubular epithelial cells. However, the increase in netrin-1 secretion was not associated with an increase in netrin-1 mRNA levels, suggesting that the regulation is at posttranscriptional levels. MAP kinases, particularly ERK and p38 MAPKs, mediate the actions of drugs and hypoxia on netrin-1 protein production. The MAP kinase mediated increase in netrin-1 production is not through an increase in mRNA stability, suggesting that regulation is at the level of translation. These results are relevant to the pathogenesis of acute kidney injury and chronic kidney disease since netrin-1 is used as an early diagnostic biomarker and has also been shown to play an important role in the pathophysiology of acute and chronic kidney diseases. Identification of the proteins involved in netrin-1 mRNA translation and the sequences to which they bind will require further investigation.
